# Atypical Presentation of Posterior Reversible Encephalopathy Syndrome (PRES): A Case Report and Review of the Literature

**DOI:** 10.7759/cureus.65290

**Published:** 2024-07-24

**Authors:** Ankit Sharma, Nidhi Kaeley, Achal Goindi, Minanshu Mittal, Jitendra K Yadav

**Affiliations:** 1 Emergency Medicine, All India Institute of Medical Sciences, Rishikesh, Rishikesh, IND; 2 Radiodiagnosis, All India Institute of Medical Sciences, New Delhi, New Delhi, IND; 3 Anesthesiology, All India Institute of Medical Sciences, Rishikesh, Rishikesh, IND

**Keywords:** periodic alternating nystagmus (pan), abnormal eye movement, periodic alternating gaze, emergency medicine, nystagmus, pres (posterior reversible encephalopathy syndrome)

## Abstract

Posterior reversible encephalopathy syndrome (PRES) is a reversible clinico-radiological entity characterized by acute neurological symptoms and white matter vasogenic edema that commonly affects the posterior occipital and parietal lobes of the brain. Patients with this condition usually present with complaints of headache, encephalopathy, seizures, or visual disturbances. Nystagmus and periodic alternating gaze are rarely reported presentations of PRES patients. Similarly, involvement of the brainstem, cerebellum, basal ganglia, and other cerebral areas are atypical findings on brain imaging. Early diagnosis and immediate treatment can reverse both the clinical and radiological features of PRES.

## Introduction

The posterior reversible encephalopathy syndrome (PRES) was first described by Hinchey in 1996 as a reversible posterior leukoencephalopathy syndrome [1]. This neurological syndrome manifests as headache, confusion, altered mental status, seizures, cortical blindness, lethargy, and stupor, and is occasionally accompanied by focal neurological signs [2]. Nystagmus and periodic alternating gaze deviation are rarely reported in PRES [3,4]. Radiologically, it appears as edema involving the white matter of cerebral hemispheres in the posterior regions, mainly the parieto-occipital lobes bilaterally. Involvement of the brainstem, cerebellum, basal ganglia, spinal cord, and other cerebral areas are atypical findings [5,6].

It has been observed that PRES is usually associated with conditions such as renal failure, hypertensive crisis, sepsis, cytotoxic drugs, autoimmune disorders, and preeclampsia or eclampsia, as all these conditions enhance acute endothelial injury with a breakdown of the blood-brain barrier (BBB), which leads to brain edema in patients with PRES [7]. As the name suggests, the condition is clinically and radiologically reversible if diagnosed promptly and treated immediately. However, a few studies reported the non-reversibility of clinico-radiological features and atypical presentation of features [8]. Here, we report a patient with PRES who presented with nystagmus and periodic alternating gaze deviation.

## Case presentation

A 40-year-old male patient presented to the emergency medicine department with complaints of abnormal eye movement and gaze deviation, followed by confused behavior for one day. He had horizontal nystagmus with alternate gaze deviation every two to three minutes (Video 1).

**Video 1:** Abnormal eye movements in the PRES patient Horizontal nystagmus was seen on ocular examination. His gaze was changing from the right side to the left side and vice-versa after every two to three minutes. PRES: posterior reversible encephalopathy syndrome

On arrival, his blood pressure was 234/150 mmHg, heart rate 70/min, respiratory rate was 20/minute, oxygen saturation was 100% on room air, Glasgow coma scale was 15/15, RBS was 150 mg/dl, and there was no focal neurological deficit. He had a history of type 2 diabetes mellitus for 15 years and was currently on regular insulin. He had chronic kidney disease for two years and was on maintenance hemodialysis three times weekly. He also had hypertension for two years and was on oral amlodipine and clonidine. He had a history of non-compliance with oral antihypertensives for two days, and his last hemodialysis was performed two days ago. There was no history of head trauma, seizure, focal neurological deficit, fever, shortness of breath, or any other past illness or medication.

On neurologic examination, there was no neck stiffness, Kernig’s and Brudzinski’s signs were negative, and no limb weakness was observed. On fundus examination, mixed retinopathy (hypertensive and diabetic) was noticed. Vestibulo-ocular reflex testing with fast head rotation was performed but gaze deviation was not resolved. Spontaneous saccades were absent. Cardiac, respiratory, and abdominal examinations were also unremarkable. Thorough hematological and biochemical investigations were done. His renal function studies were deranged with raised serum urea and creatinine values (Table 1).

**Table 1:** Kidney function test at presentation

Kidney Function Test	Result	Unit	Reference Range
Serum Urea	112	mg/dL	15-45
Serum Creatinine	4.1	mg/dL	0.6-1.3
Serum Sodium	135	mmol/L	135-155
Serum Potassium	3.8	mmol/L	3.5-5.5
Serum Calcium	8.8	mg/dL	8.5-10.5
Serum Uric Acid	5.2	mg/dL	3-7.6

**Figure 1:**
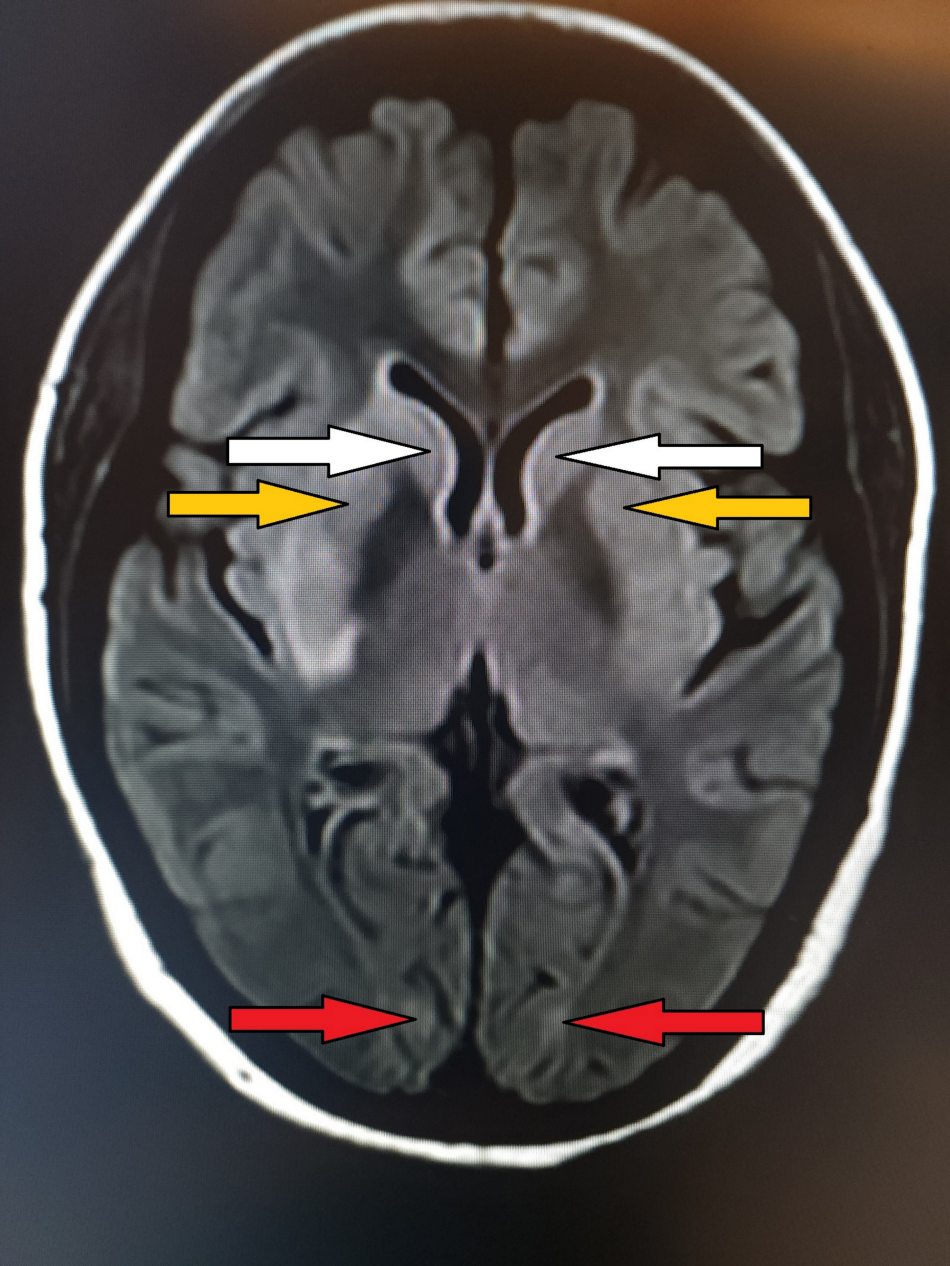
MRI brain (FLAIR) showing bilateral hyperintense lesions at multiple sites. White arrows show hyperintensities in the head of the caudate nucleus bilaterally; Yellow arrows show hyperintensities in the putamen region bilaterally; Red arrows show hyperintensities in the occipital region bilaterally. MRI: magnetic resonance imaging; FLAIR: fluid-attenuated inversion recovery

**Figure 2:**
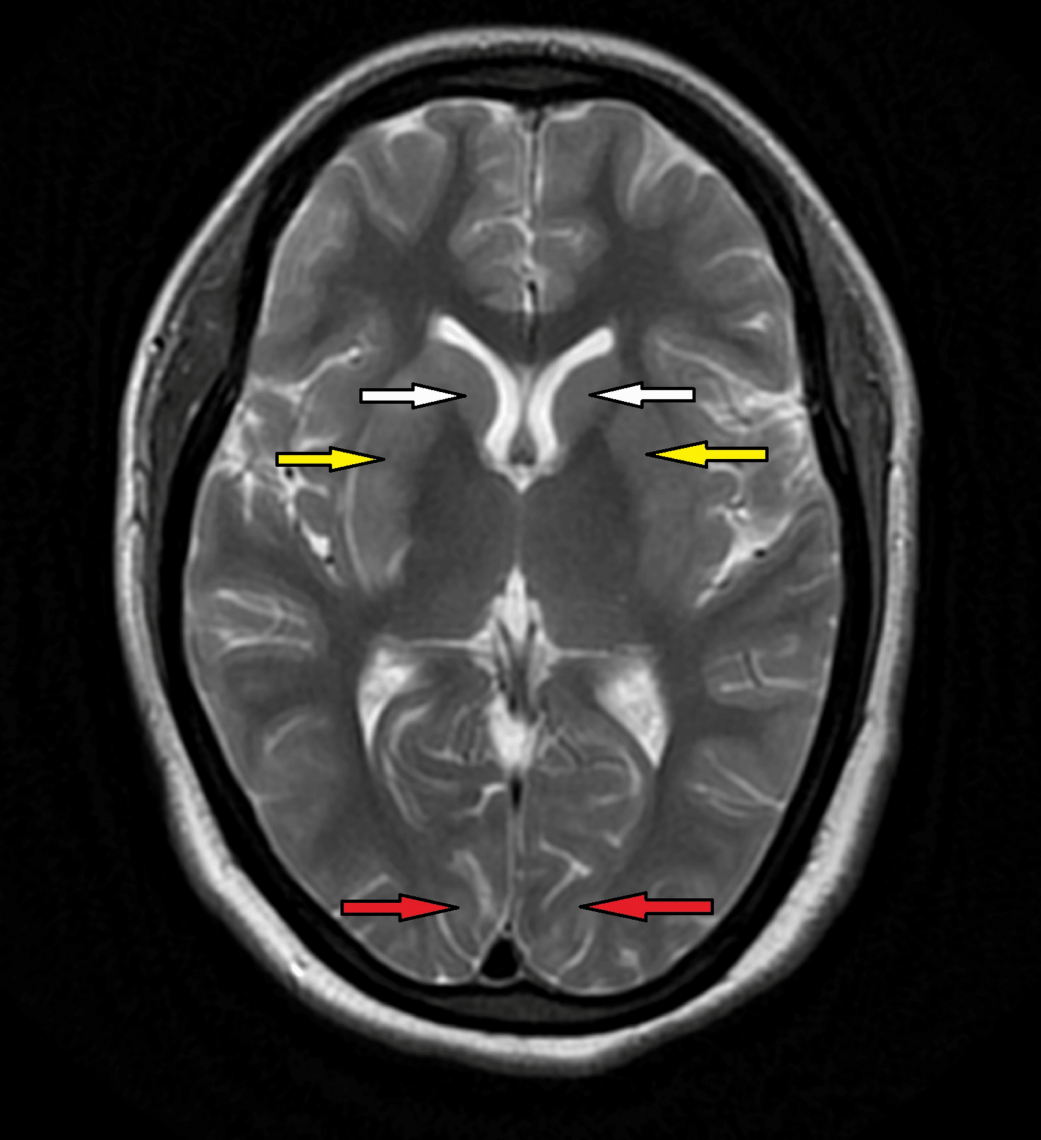
MRI brain (T2W) showing bilateral hyperintense lesions at multiple sites. White arrows show hyperintensities in the caudate nucleus bilaterally; Yellow arrows show hyperintensities in the putamen region bilaterally; Red arrows show hyperintensities in the occipital region bilaterally. MRI: magnetic resonance imaging; T2W: T2 weighted image

**Figure 3:**
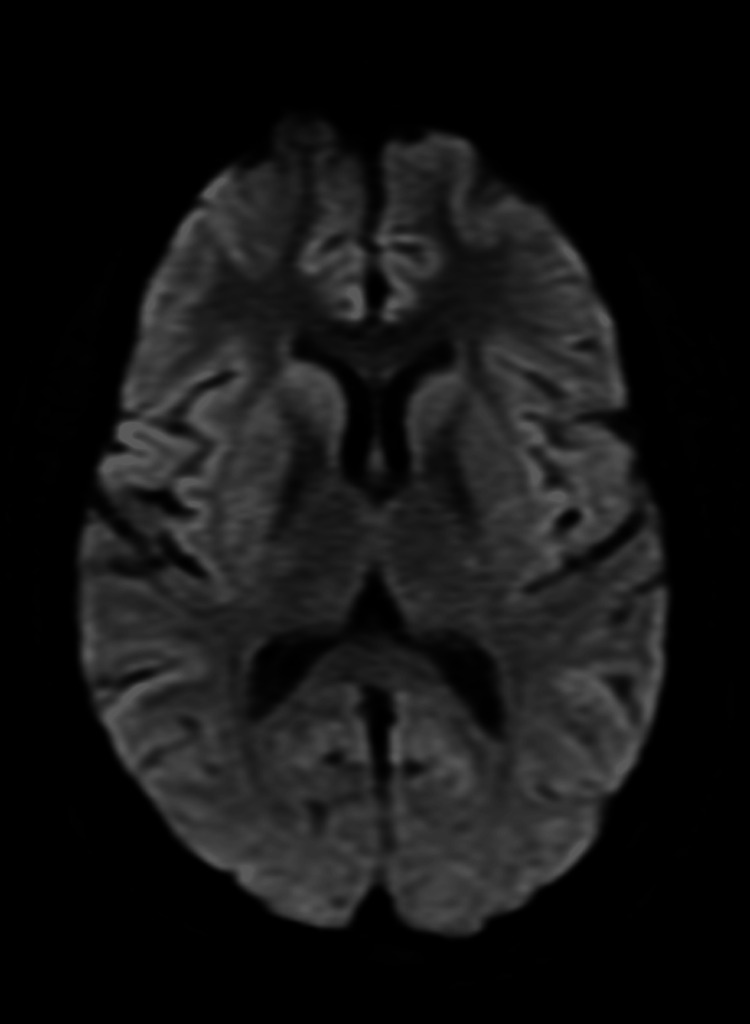
MRI brain (DWI) showing no restricted diffusion in the basal ganglia and occipital region. MRI: magnetic resonance imaging; DWI: diffusion-weighted imaging

**Figure 4:**
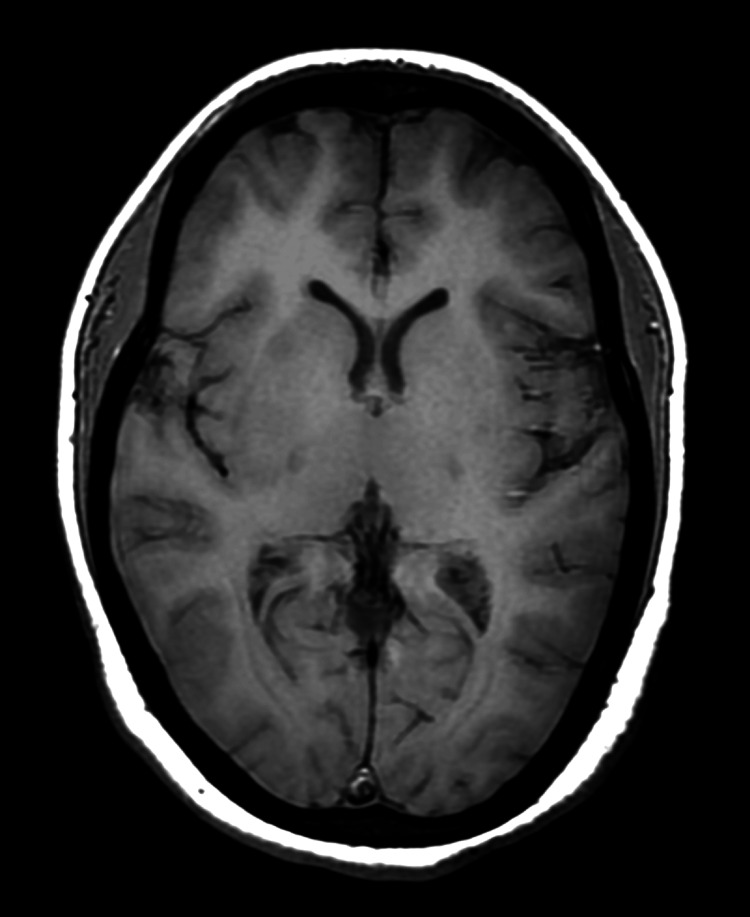
MRI brain (T1W) showing bilateral isointense signals in bilateral basal ganglia and occipital regions. MRI: magnetic resonance imaging; T1W: T1 weighted

There was no evidence of electrolyte imbalance as potassium, magnesium, or sodium levels were within normal limits. His hematological investigation revealed mild anemia (hemoglobin 9 g/dl). Blood cell count, liver function test, procalcitonin, serum glucose levels, thyroid profile, and creatine phosphokinase (CPK) were all within normal limits. CSF analysis was also within normal limits. His reverse transcriptase-polymerase chain reaction (RT-PCR) swab for COVID-19 was also sent, which later came out negative. EEG was also performed for the patient to rule out any seizure activity, but the results were consistent with encephalopathy. Brain MRI showed hyperintensities in the basal ganglia (putamen and caudate nucleus) and occipital region bilaterally on the fluid-attenuated inversion recovery (FLAIR) image (Figure 1). Hyperintensities in the basal ganglia (putamen and caudate nucleus) and occipital region bilaterally were seen on the T2-weighted (T2W) image also (Figure 2). On diffusion-weighted imaging (DWI), no restricted diffusion was seen in the basal ganglia and occipital regions (Figure 3), and the T1-weighted (T1W) image showed isointense signals in bilateral basal ganglia and occipital regions (Figure 4).

He was started on antihypertensives including, intravenous nicardipine, oral clonidine, and amlodipine. Hemodialysis was also performed on the day of the presentation. Following this, his blood pressure decreased to 190/120 mmHg. On the second day, his antihypertensives were continued and improvement in nystagmus and gaze deviation was noted. After 36 hours of presentation, his blood pressure was recorded as 160/100 mmHg, with complete resolution of symptoms. There was no recurrence of symptoms noted later on.

**Figure 5:**
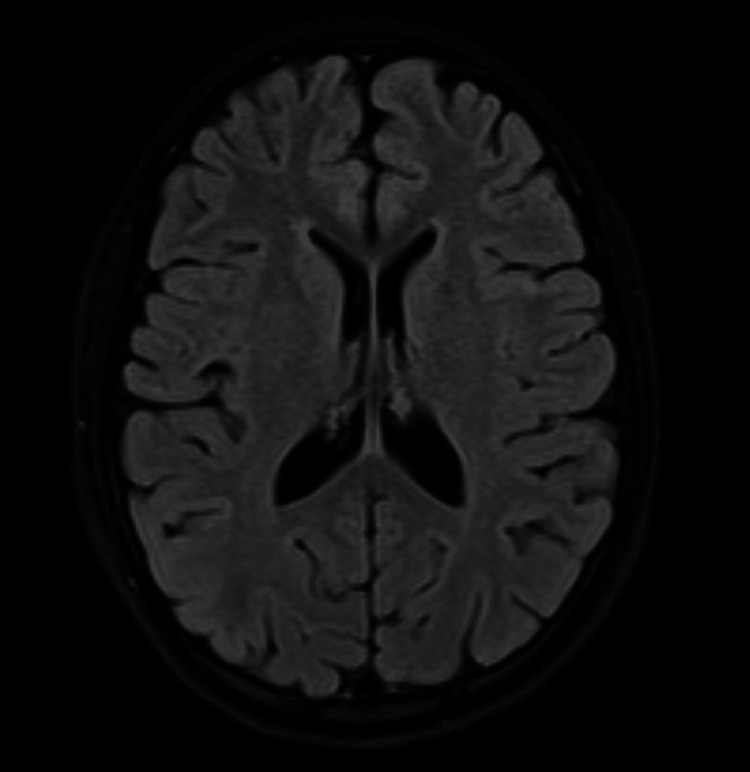
MRI brain (FLAIR) at the one-month follow-up shows resolution of the lesions in the basal ganglia and occipital region. FLAIR: fluid-attenuated inversion recovery

These acute onset and reversible neurological symptoms along with symmetrical vasogenic edema on brain MRI were suggestive of PRES [9]. He was given three hemodialysis sessions during his hospital stay and was, discharged after one week in a symptom-free state. The patient was followed up after one month of discharge. He was taking his antihypertensive and oral hypoglycemic drugs regularly along with hemodialysis sessions thrice weekly. He was completely asymptomatic and was performing all his daily activities. Follow-up MRI brain also showed resolution of the lesions in the basal ganglia and occipital region (Figure 5).

## Discussion

The term PRES justifies its name based on the clinical and radiological reversibility of features and involvement of the posterior part of the brain (parieto-occipital lobes), commonly. Three hypotheses have been proposed to explain the pathophysiology of PRES: (i) Cerebral vasoconstriction causing subsequent infarcts in the brain, (ii) failure of cerebral autoregulation with vasogenic edema, and (iii) endothelial damage with BBB disruption leading to fluid and protein transudation in the brain. However, the exact mechanism of injury is still not clear [10].

Patients commonly present with headache, confusion, altered mental status, seizures, cortical blindness, lethargy, stupor, and occasionally focal neurological signs [11]. Periodic alternating nystagmus (PAN) and periodic alternating gaze deviation (PAGD) are rare symptoms of PRES. PAN is defined as horizontal jerk nystagmus present in the primary position of gaze and is characterized by an alternation of direction to the right and then to the left, with a nystagmus-free interval (null period) in between [12]. PAGD refers to the involuntary horizontal conjugate deviation of the eyes, with the direction alternating approximately every one to two minutes; therefore, PAGD can be considered a variant of PAN, as fast-phase eye movements are impaired or absent in it due to concomitant dysfunction of brainstem saccade generators. The underlying pathophysiological mechanism for both PAN and PAGD is the dysfunction of vestibulocerebellar pathways, which may destabilize the velocity storage mechanism of the central vestibular system that regulates the temporal course of normal rotationally induced nystagmus [3,13-16]. This can explain the presentation of our patient with lateral gaze and alternating nystagmus simultaneously. MRI brain images of our patient showed involvement of the basal ganglia and occipital regions. This evident edema of parts of the basal ganglia in our patient can cause PAGD and PAN. Moreover, edema of other parts (i.e. brainstem, etc.) below the threshold of radiographic detection could not be excluded. This patient was a known case of hypertension and renal failure which are common risk factors for PRES. Other risk factors include preeclampsia/eclampsia, autoimmune diseases, infection, transplantation, and the use of chemotherapeutic agents [17]. Additionally, an association between COVID-19 infection and PRES has been reported previously [18].

Mackay et al. reported a similar case in 2014, in which a 73-year-old man presented with obtundation, PAGD, intermittent nystagmus, and markedly elevated blood pressure [3]. On MRI, vasogenic edema was seen in the cerebellum and temporo-occipital lobes, indicative of PRES. Intravenous nicardipine was administered to normalize his blood pressure. His symptoms resolved in 48 hours, and he was discharged on day 7. His MRI showed resolution of edema after two months.

The mainstay treatment for PRES targets the risk factors such as hypertension and renal failure. For lowering blood pressure, intravenous antihypertensives are preferred, and an initial reduction of 20% seems reasonable [19]. For fluid overload, hemodialysis should be considered for such patients [20]. In our patient, the presenting features were PAGD, PAN, and confused behavior, which are the atypical presentations of PRES. MRI brain showed hyperintensities in the basal ganglia along with the occipital region, which justifies this atypical presentation of PRES. Immediate intervention in the form of blood pressure control and hemodialysis led to complete resolution of symptoms in 36 hours, with reversal of radiological changes at the one-month follow-up imaging. Based on previous literature, the mean time to full clinical recovery in PRES patients ranges from two to eight days [7]. Reversal of radiological findings has been noted in follow-up imaging, with an average follow-up time ranging from one to two months in the majority of studies [3,9]. So early detection and immediate intervention are the treatment goals of PRES, and long-term management can prevent relapse.

## Conclusions

PAN and PAGD are rarely reported symptoms of PRES. Involvement of the atypical sites in the brain can cause such symptoms in these patients. Early detection and intervention can reverse the clinical and radiological features of PRES. Knowing about this rare and atypical clinico-radiological presentation of PRES can help improve the diagnosis and treatment of these patients.
